# The 8^th ^annual computational and systems neuroscience (Cosyne) meeting

**DOI:** 10.1186/2042-1001-1-8

**Published:** 2011-04-20

**Authors:** Mark H Histed, Jonathan W Pillow

**Affiliations:** 1 Department of Neurobiology, Harvard Medical School, Boston, USA; 2 Departments of Psychology and Neurobiology, Center for Perceptual Systems, The University of Texas at Austin, Austin USA

## Overview

The 8^th ^annual Computational and Systems Neuroscience meeting (Cosyne) was held February 24-27, 2011 in Salt Lake City, Utah (abstracts are freely available online: http://www.cosyne.org/c/index.php?title=Cosyne2011_Program). Cosyne brings together experimental and theoretical approaches to systems neuroscience, with the goal of understanding neurons, neural assemblies, and the perceptual, cognitive and behavioral functions they mediate.

The range of questions available to systems and computational neuroscience has grown substantially in recent years, with both theoretical and experimental approaches driven by the increasing availability of data about neural circuits and systems. The Cosyne meeting has reflected this growth, nearly doubling in size since the first meeting in 2004, to a new record of nearly 600 attendees this year. It remains single-track, which allows discussions of presentations to drive scientific interaction between attendees with diverse backgrounds. Poster sessions take place each evening, which provide a forum for intense scientific conversations that frequently spill out into more informal settings late at night. The meeting is followed by two days of workshops, held at the Snowbird ski resort, which feature more specialized talks and interactive discussions on a wide collection of topics, this year ranging from consciousness and compressed sensing to dynamics, learning, and perception.

**Figure 1:**
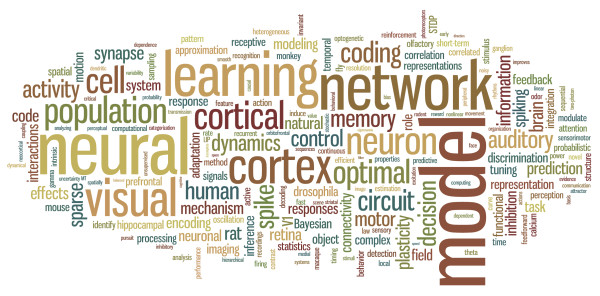
**Word cloud of abstract keywords at Cosyne 2011**. In this diagram, word size reflects rate of incidence of the word in this year's abstracts. (Created by Eero Simoncelli, NYU, using the software at http://wordle.net)

We observed a few major emerging themes. The focus on neural circuits is clear; many investigators are using detailed knowledge of anatomy, including cell identity and network connectivity, to understand neural activity and function. Several model systems for studying circuits received major focus, including the fly, zebrafish, rat, and mouse. An important strength of these systems is the ability to manipulate circuits genetically, and studies with genetic components generated significant enthusiasm. A principal question remains how neural activity relates to behavior, with the number of studies in the above model systems increasing, alongside continued behavioral work in humans and non-human primates. Functional coupling between neurons is a key topic of interest; many presentations addressed methods and theories for understanding the impact of coupling on computation and network function, and we predict these efforts will only grow in future years. Questions of coupling in time, such as oscillatory activity, continued to attract considerable attention. The interaction of excitatory and inhibitory influences has implications for many neural circuits, and a diverse set of theoretical studies explored the implications of the 'balanced' state for computation and information transmission. Comparatively little experimental data on inhibitory and excitatory interactions exists and we predict there will be an upswing of experiments studying these dynamics in the next few years. Bayesian statistical theories continued to play a major role, both as methods for analyzing neural data and as theories for optimal information processing in perceptual and motor tasks. Finally, sensory systems have historically been strongly represented in systems and computational neuroscience. While this continued, there was broadening interest in motor systems and the representations and computations underlying movement. The topics covered by submitted abstracts were summarized nicely in a single slide (Figure 1) presented by Anne Churchland (Cold Spring Harbor), co-chair of the organizing committee.

Below we highlight a few presentations of special interest. We have made an effort to sample broadly, but Cosyne appeals to a large audience across several disciplines, and we are limited by space and a residual slant towards our own interests and interactions at the meeting. We apologize to those presenters whose contributions we do not have space to mention, but we are excited about the broad extent of new work we observed.

### Neural activity and perception

Stanislas Dehaene (INSERM/CEA) gave a wide-ranging summary of his research on how humans perceive and process numerosity, citing behavioral studies of infants and diverse human societies, and connecting that to neural findings in macaques. Jonathan Victor, D. Thengone, and M. Conte (Weill Cornell Medical College) presented a novel approach for characterizing the perceptual salience of low and high-order statistics in natural images using an innovative method for parameterizing textures; their findings suggested that image statistics interact perceptually according to an approximately Euclidean distance function. Wilson Geisler (UT Austin) showed another method for exploiting higher-order statistical properties of natural images using local measurements from a very large collection of images. These statistics were then used to derive Bayesian solutions to a variety of low-level vision problems. Saskia de Vries and T. Clandinin (Stanford) identified a group of *Drosophila *visual neurons that detect objects on a collision course with the fly; inactivating these prevents the animal from moving to avoid a collision.

### Circuits affecting behavioral computations

Tirin Moore (Stanford) described new work linking the dopamine circuits of the frontal cortex of the macaque both to activity of visual neurons and to behavioral responses. David Anderson (Caltech) described beautiful work dissecting circuits in the ventromedial hypothalamus that underlie aggression in the mouse. Inducing spiking activity in a small but specific population of neurons in the amygdala produces a complex, sustained attacking behavior in which male mice are induced to attack females, a rare event in their normal behavior. Franz Weber, C. Machens, and A. Borst (MPI of Neurobiology) presented an elegant application of the generalized linear model (GLM) to functional coupling between two identified fly neurons (H1 and Vi) implicated in the processing of visual motion. Their analyses revealed a unidirectional coupling from H1 to Vi, tuned to produce optimally informative representations in Vi. Finally, Surya Ganguli (UCSF) and R. Hahnloser showed that local, Hebbian learning rules can explain rapid learning of complex sequences by neural circuits, a novel paradigm for sequence learning that poses a significant theoretical challenge to reinforcement learning models. The range of this work highlights the interest in coupling between neurons; we believe a major goal for the field is to determine which behaviors rely on a small number of neurons, and which are more sensitive to coupling due to the dynamic interaction of many neurons or multiple circuits.

### Information processing in neural assemblies

E.J. Chichilnisky (Salk) reviewed his work identifying the functional connectivity between individual cones and ganglion cells in the primate retina and computational methods to infer bipolar cell connectivity. Alison Barth (Carnegie-Mellon) discussed a series of studies on subsets of highly-active neurons in the mouse cerebral cortex and how they might affect the computations performed there. Murray Sherman (Univ. of Chicago) presented an alternative to the usual view that representations are constructed sequentially (for example, first by the sensory thalamus, then primary, and then secondary sensory cortex and so on). He outlined how subcortical regions, including the thalamus, might support more parallel or simultaneous processing. Elad Ganmor, R. Segev, and E. Schneidman (Weizmann Inst.) described a novel approach for capturing the joint activity of very large populations of neurons using sparse, low-order interaction networks. Brice Bathellier and S. Rumpel (IMP Vienna) used two-photon calcium imaging in mouse auditory cortex to show that neural subpopulations can combine to represent a large number of diverse sounds and also predict performance in a sound discrimination task. Rubén Moreno-Bote and A. Pouget (Rochester) used an analysis of spiking neural networks to argue that decorrelation does not affect the amount of information available to downstream populations, thus calling into question a central dogma of population coding.

### Understanding network structure

Tony Zador (Cold Spring Harbor Laboratory) discussed a new method for solving a major challenge facing the field - determining which neurons are connected to each other. The method exploits the tremendous advances in DNA sequencing technology. It uses short oligonucleotides to uniquely tag neurons, and viral machinery to transport the tags across synapses where they are identified via sequencing. Ian Ellwood and V. Sohal (UCSF) used both experiments and models to show how dopaminergic inputs can strongly modulate cells' firing through intertwined effects on calcium, potassium, and sodium channels. Sandra Kuhlman, E. Tring, and J. Trachtenberg (UCLA) showed that mouse visual inhibitory neurons acquire broader visual tuning during development, though excitatory neurons sharpen their tuning as a result of activity. John Cunningham (Cambridge), M. Churchland, M. Kaufman and K. Shenoy presented 'jPCA', a method for reducing the dimension of large neural datasets by looking for rotational or oscillatory dynamics. Mark Churchland, J. Cunningham, M. Kaufman, S. Ryu and K. Shenoy (Stanford) showed an application of this method to unit recordings from macaque motor cortex and argued that slow (1-3 Hz) network oscillations seem to be an important basis for motor control.

The field of computational and systems neuroscience is advancing quickly, driven both by innovation in experimental approaches and simultaneous development of theoretical ideas to understand these data. The growth and energy of the Cosyne meeting clearly reflect both trends.

## Competing interests statement

The authors declare they have no competing financial interests. JWP has collaborated with E.J. Chichilnisky.

